# Missouri Valley Veterinary Association

**Published:** 1895-04

**Authors:** S. L. Hunter

**Affiliations:** Secretary and Treasurer


Secretary.

### MISSOURI VALLEY VETERINARY ASSOCIATION.

THE fourth regular meeting of the Missouri Valley Veterinary Association was held in Topeka, Kansas, February 13, 1895, and distanced all other meetings of the Association in numbers and interest. The following gentlemen were present: Drs. Stewart and Barth, Kansas City, Kas.; Wattles, of

Kansas City, Mo.; Moore and Linscott, of Holton, Kas.; Brady, Mayo, and Orr, Manhattan, Kas.; Cook, of Hutchinson, Kas.; Richards, of Emporia, Kas.; McCurdy, Prichard, and Knisely, Topeka, Kas.; McClellan, Lawrence, and Verschelden, St. Mary's, Kas.; Hunter, of Fort Leavenworth, Kas. Messrs. Topping and Stalker, veterinary students of the Kansas City Veterinary College, and Messrs. Brown and White, of the Live Stock Sanitary Board, were present. Letters from Drs. Howard, McCasey, Harrison, Biart, and others were read, regretting their inability to attend.

The Committee on Legislation reported, and were directed to continue in the same capacity.

It was the opinion of all that the Live Stock Sanitary Board should have control of hog cholera and swine plague, and the power to quarantine, which could be done by the Legislature rescinding Section 4411, Laws of 1884; Messrs. White and Brown making timely and valuable suggestions thereon.

The proper disinfection of railroad cars was also considered.

Dr. Mayo's paper on "Cattle Poisoning by Cornstalks" was listened to with great interest, and elicted much discussion.

The stereopticon was used to illustrate many points in meat inspection, which was new to many members.

Dr. Harrison's paper on "Operation for Periodic Ophthalmia" was defended by President Stewart in a most able manner.

Our membership is now thirty, and still growing.

The following resolution was read and unanimously adopted:

*Resolved*, That this Association condemn the action of so-called veterinary dentists or veterinary surgeons in teaching that branch of veterinary science consisting of dental operations upon horses' teeth, castration, and spaying, or any other branch thereof, to men that are not graduates of regular veterinary colleges, as we believe it increases the number of unqualified practitioners, and tends to lower the standard of this profession; and that teaching such men veterinary dentistry or any part of veterinary science by any member of this Association shall be considered as a breach of the Code of Ethics.

Drs. Black, Sihler, Linscott, Brady, Barth, Ernst, and Forbes were appointed by President Stewart to furnish papers for our annual meeting, which will be held at Leavenworth, in June.

The Association gratefully acknowledges the kindness of the professional brethren of Topeka, for entertainment during its stay in Topeka.

S. L. Hunter, V.S.,
Secretary and Treasurer.

